# Early Onset Immune-Related Adverse Event to Identify Pseudo-Progression in a Patient With Ovarian Cancer Treated With Nivolumab: A Case Report and Review of the Literature

**DOI:** 10.3389/fmed.2020.00366

**Published:** 2020-07-28

**Authors:** Hui Li, Xin Zhou, Ding Zhang, Guoqiang Wang, Xiaochun Cheng, Caihong Xu, Bin Yao, Linrong Pang, Jun Chen

**Affiliations:** ^1^Department of Radiotherapy and Chemotherapy, The Affiliated People's Hospital of Ningbo University, Ningbo, China; ^2^The Medical Department, 3D Medicines Inc., Shanghai, China

**Keywords:** immunotherapy, ovarian cancer, nivolumab, biomarker, pseudo-progression

## Abstract

**Background:** Immune checkpoint inhibitors (ICIs) have shown clinical benefit in many advanced tumors, however, pseudo-progression is a noted phenomenon of ICIs characterized by radiologic enlargement of the tumor burden, followed by regression. How to differentiate pseudo-progression from progression remains a critical clinical issue. Recent studies have demonstrated the association between immune-related adverse events (irAEs) and efficacy of ICIs. Here we demonstrated an ovarian cancer patient treated with nivolumab in whom the early-onset irAE was identified to differentiate pseudo-progression from progression.

**Case presentation:** Here we present the case of a 47-years-old woman with histopathologically confirmed diagnosis of metastatic ovarian cystadenocarcinoma. Immunohistochemical examination showed 10% of tumor cells to express the programmed cell death receptor ligand 1 (PD-L1) and a 381-gene panel sequencing in a College of American Pathologists (CAP) certificated lab revealed a moderate mutational tumor burden with 5.7 Mutants/Mb. The patient received nivolumab 100 mg every 2 weeks as a fourth line treatment. Within the first 2 months, the tumor volume increased by 23.6%. However, the patient experienced an elevation of Alanine aminotransferase (ALT) and Aspartate aminotransmerase (AST), which was diagnosed as the immune-related hepatitis. Thus, the treatment continued and afterwards, the patient reached a partial response (PR) to nivolumab and the progression-free survival was 9 months.

**Conclusion:** To our knowledge, this is the first case describing early-onset immune-related adverse events to identify pseudo-progression in a patient with ovarian cancer treated with nivolumab, and PD-L1 expression level may be a predictive biomarker in the immunotherapy of ovarian cancer.

## Background

In the past decades, the immune checkpoint inhibitors (ICIs) have provided a new therapeutic strategy for cancer patients with promising activity and manageable toxicity (1). Immunotherapy has become a standard of care for multiple cancer types including non-small-cell lung cancer, melanoma, renal cell carcinoma and bladder cancer (2). During the ICIs therapy, pseudo-progression is a special response pattern in patients who experienced delayed tumor regression after initial radiographic progression. Pseudo-progression was first found in the research of melanoma with the anti-cytotoxicT-lymphocyte-associatedprotein 4 (CTLA4) inhibitor (3), then in the studies of programmed cell death receptor 1 (PD-1) antibody including nivolumab and pembrolizumab (4). In the clinical trials, the incidences of pseudoprogression are 0.6–5.8, 1.5–7.1, 6.9, and 1.1% in non-small-cell lung cancer, urothelial carcinoma, mesothelioma, and Merkel cell carcinoma, respectively. In general, pseudo-progression is identified by tumor burden increase pathologically and performance status of patients (5). However, radiological evidence and physical condition are not sufficient for clinicians to differentiate pseudo-progression from real progression.

ICIs can lead to loss of self-tolerance and increased activation of T-effector cells, resulting in tumor cell death. The loss of self-tolerance also leads to autoimmune cytotoxicities, named immune-related adverse events (irAEs). Overall, the incidence of irAEs with anti-PD-1 treatments is ~25% (6). Common irAEs include dermatitis and thyroiditis. Less common but potentially more serious irAEs include pneumonitis, colitis, hepatitis, nephritis, hypophysitis, adrenalitis, and myositis (7). irAEs are beyond common side effects that require treatment, they also open windows for clinicians to predict the efficacy of ICIs. Recent studies have demonstrated the association between irAEs and improved clinical outcomes in various tumor types (8–13). In non-small cell lung cancer (NSCLC) treated with nivolumab, the progressive-free survival (PFS) and overall survival (OS) was longer in patients with irAE occurring within 6 weeks post-treatment than those without irAE (9).

Thus early onset irAEs may also provide a tool to differentiate pseudo-progression from progression. However, whether early onset irAEs could be used to identify pseudo-progression remains to be studied.

## Case Presentation

In this case, a 47-years-old woman patient was admitted to Ningbo Women and Children's hospital due to the sustaining fever, fatigue, abdominal bulge, chest distress and short breath in September, 2018. The patient was previously diagnosed with ovarian cystadenocarcinoma associated with omental metastasis. Before here, the patient underwent transabdominal epifascial hysterectomy, double adnexectomy, greater omentectomy, appendicectomy, and cytoreductive surgery, then after relapse arboplatin/cisplatin plus paclitaxel, albumin paclitaxel plus bevacizumab and gemcitabine plus bevacizumab were treated in turn. However, the patient was less able to tolerate the chemotherapy. In our department, the radiological images showed post-operative changes of ovarian cancer, including multiple soft tissue masses in pelvis, the localized thickening of the intestinal wall and soft tissue nodules in the lower abdominal wall.

To seek for other potential therapeutic opportunities, the patient received a next generation sequencing (NGS) using a 381-gene panel performed in a CAP-certificated lab and immunohistochemistry (IHC) detection of PD-L1 expression during symptomatic support treatment. NGS results indicated that the tumor mutation burden (TMB) was 5.65 Mutants/Mb and IHC results indicated that the fraction of tumor cells that expressed PD-L1 was around 10%.

**Figure 1:**
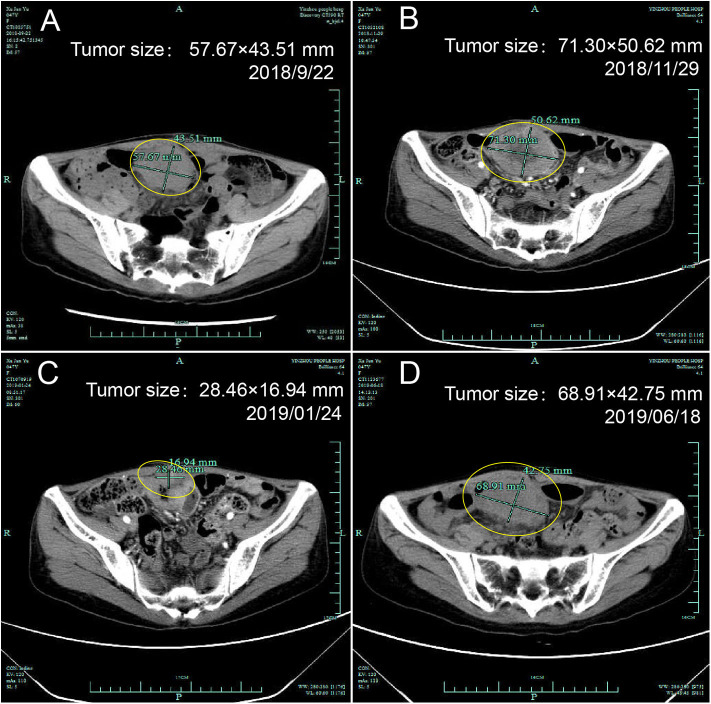
Computed tomography of the ovary before and after initiation of nivolumab treatment. **(A)** Tumor status before treatment, **(B)** Pseudo-progression within the first 2 months of treatment, **(C)** Partial response after treatment, **(D)** Progression.

**Figure 2:**
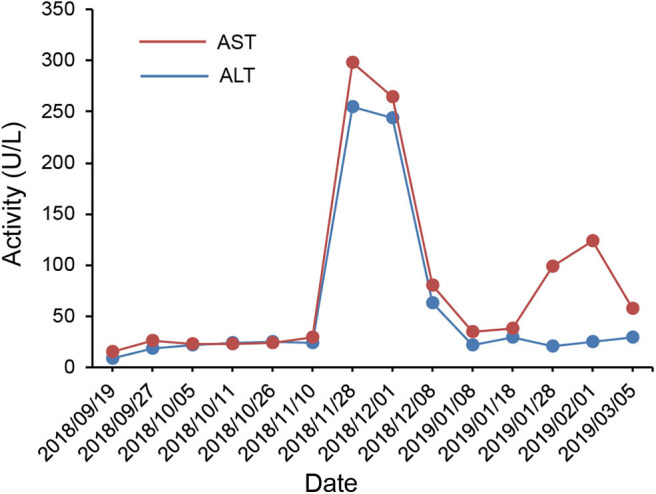
Course of aspartate aminotransferase (AST) and alanine aminotransferase (ALT) in serum in relation to applied therapies; course of biochemical markers over time.

**Figure 3:**
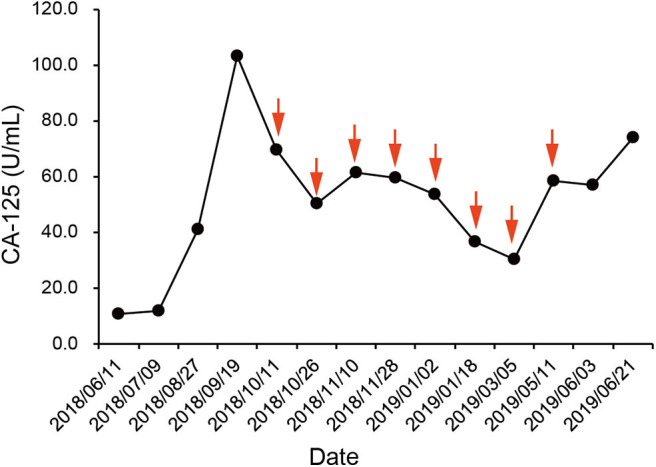
Course of cancer antigen 125 (CA-125) levels in relation to applied therapies; course of tumor markers over time. Arrows indicate application of nivolumab therapy.

According to the IHC results, the patient received nivolumab therapy 100 mg every 2 weeks since September, 2018. Within the first 2 months of treatment, computed tomography (CT) results showed that the tumor size was increased from 57.67 × 43.51 to 71.30 × 50.62 mm (Figures 1A,B) and an unconfirmed progression was observed according to Immune Response Evaluation Criteria in Solid Tumors (iRECIST) criteria (14). However, the patient experienced an elevation of Alanine aminotransferase (ALT) and Aspartate aminotransmerase (AST) (Figure 2), which was diagnosed as the immune-related hepatitis according to CTCAE grading system (15). According to literature research showing the association between irAEs and efficacy, the presence of liver irAE might suggest that the patient might be benefiting from immunotherapy even though the tumor was enlarged. So we considered that the patient might experience a pseudo-progression and continued the treatment of nivolumab. Also the tumor biomarker cancer antigen 125 (CA-125) dropped from 103.1 to 50.2 U/mL (Figure 3).

In January 2019, after 4 months treatment of nivolumab, the fatigue symptom of the patient was dramatically improved, and the tumor size was decreased with a shrinkage of about 50.7% compared with the size before the treatment. It was concluded that the patient reached a partial response (PR) (Figure 1C). The CA-125 remained plateau during the treatment and increased on June 21st (Figure 3), in accordance with the increase of tumor size in the CT results (Figure 1D). The progression-free survival was about 9 months.

Regarding side effects, the patient experienced a treatment related fever with 40.2°C after the first administration. By physical cooling, the body temperature returned to normal gradually. On November 28th, 2018, methylprednisolone (40 mg, every 12 h) was injected intravenously to treat the hepatitis. The ALT and AST gradually returned to normal (Figure 2). In general, the side effects of nivolumab were manageable.

## Discussion

Pseudo-progression is defined by radiologic enlargement of the tumor burden, followed by regression (16). Patients with solid tumors may be accompanied with pseudo-progression during the immunotherapy. Hodi et al. reported that 4 of 107 patients with metastatic melanoma treated with nivolumab had an unconventional response patterns indicative of pseudo-progression (17). Another study found that 2 of 41 patients with non-small-cell lung cancer treated with ICIs were classified as having radiologic progression initially and then experienced tumor regression (18). Although the underlying mechanism of pseudo-progression is unclear, immunocyte infiltration may be involved in the formation of pseudo-progression, which result in the enlargement of tumors.

How to distinguish pseudo-progression from progression of immunotherapy has drawn great attention of clinicians. However, there is no good solution to this problem. In the study by Nishino et al., three advanced melanoma patients treated with pembrolizumab had target lesion increase with subsequent response. Before delayed tumor shrinkage, all these patients experienced two or more consecutive radiologic scans during the timeframe of minimum 4 weeks, thus confirming progression disease (19). Therefore, imaging examinations alone might not be enough to identify pseudo-progression and could be risky for patients.

In our report, we used early-onset irAEs as a predictor to differentiate psedo-progression to progression. The appearance and development of irAEs strongly correlates with survival benefit. In Osorio's cohort, thyroid dysfunction during pembrolizumab treatment of NSCLC was common and was characterized by early-onset (10/51, 21%). Ten patients (21%) with NSCLC treated with pembrolizumab developed thyroid dysfunction. In patients who developed thyroid dysfunction, the median OS on pembrolizumab was significantly longer than those without thyroid dysfunction (8). Haratani also investigated that the irAE profile and its association with clinical activity for nivolumab in NSCLC (9). Of 134 NSCLC patients treated with nivolumab, 43 patients (32%) occurred skin irAEs, 12 patients (9%) occurred astrointestinal irAEs, 7 patients (5%) occurred hepatobiliary irAEs, and 11 patients (8%) occurred other irAEs including fatigue, appetite loss, polyarthritis, and myasthenia gravis. The results revealed that any irAEs were associated with nivolumab efficacy in patients with NSCLC. So was the results of Teraoka's study (11). The association between irAEs and survival was also previously observed in melanoma patients treated with immunotherapy. The development of cutaneous irAEs, especially of hypopigmentation in patients with melanoma, could point toward better treatment response. A statistically significant OS difference was noted among melanoma patients with any grade of irAE vs. those without (12, 13). Pan-cancer analysis revealed that lichenoid and spongiotic dermatitis associated with PD-1/PD-L1 inhibitors could improve oncologic outcomes (10). However, the mechanisms underlying the association of irAEs with outcome of treatment with PD-1/PD-L1 inhibitors remains to be determined. Taken together, early-onset irAEs may be used as an indicator that patients may benefit from immunotherapy. In the case, we observed a radiologic enlargement of tumor within the first 2 months of treatment, accompanied by an elevation of AST and ALT. We considered the elevation of AST and ALT as immune-related hepatitis. The presence of immune-related hepatitis suggested that the patient might be benefiting from immunotherapy. Thus, we considered the enlargement of tumor site as pseudo-progression instead of progression and continued the treatment. After 4 months of nivolumab treatment, the patient reached a PR, further confirmed our judgment.

irAEs can occur within the first few weeks to months after treatment, even after treatment discontinuation (20). The incidence of irAEs of any grade and severe grade in patients treated with anti-PD-1/PD-L1 agents was 38.50 and 4.81%, respectively. Commonly recognized irAEs included endocrine dysfunction, diarrhea (9.47%), AST increase (3.39%), vitiligo (3.26%), ALT increase (3.14%), pneumonitis (2.79%), colitis (1.24%), bilirubin increase (1.05%), hepatitis (0.85%), and uveitis (0.29%). Among the endocrine dysfunctions, the most frequent all grade adverse events were hypothyroidism (6.07%) and hyperthyroidism (2.82%), followed by hyperglycemia (1.20%), thyroiditis (0.75%), and adrenal insufficiency (0.69%) (21).

Ovarian cancer, the second most common gynecological cancer, is the leading cause of death from gynecological cancers (22). The immune checkpoint inhibitors have not been approved by Food and Drug administration (FDA) for ovarian cancer. The study of immunotherapy in ovarian cancer is limited to clinical trials with small sample sizes. In a phase II trial of 20 patients with ovarian cancer, the ORR of nivolumab was 15%, the median OS was 20.0 months (23). In Keynote 100, the median PFS and OS were 2.1 and 17.6 months, respectively (24). In JAVELIN study, the median OS of avelumab was 11.2 months (25). Only a small subset of patients with ovarian cancer may respond to immune checkpoint inhibitors, making it of great significance to identify patients who may respond. The results were far from satisfied, thus whether nivolumab is effective in ovarian cancer and what kind of patients would respond to nivolumab need to be further studied.

Immunohistochemical examination revealed 10% of tumor cells to express the PD-L1. PD-L1 expression has been approved as a predictive biomarker in non-small cell lung cancer (NSCLC) by FDA. In KEYNOTE 042 trial, the OS was 39.3 vs. 28.0% in PD-L1+ (≥1%) vs. PD-L1– (<1%) NSCLC patients, and PD-L1+ (≥1%) patients reached a medium OS of 20 months (26). In melanoma and bladder cancer, the higher the expression of PD-L1, the greater the therapeutic effect. For ovarian cancer studies, the medium OS of avelumab was 13.8 vs. 7.0 months in PD-L1+ (≥1%) vs. PD-L1– (<1%) patients, and nivolumab reached an ORR of 15% and the median OS of 20.0 months, irrespective of PD-L1 expression (23). In this case, the patient had a positive expression of PD-L1 and reached a PR, which suggested that PD-L1 expression level may also be a potential biomarker for immune checkpoint inhibitors in ovarian cancer.

## Conclusion

In conclusion, this is the first case identifying pseudo-progression by early onset immune-related adverse event, and PD-L1 expression may be a potential biomarker for immunotherapy in ovarian cancer. Nivolumab demonstrated encouraging clinical efficacy and tolerability in an ovarian cancer patient with PD-L1 positive expression after failure of multiple lines of therapies. However, it is only a case report and the results need to be further explored with larger sample sizes.

## Ethics Statement

Ethical review and approval was not required for the study on human participants in accordance with the local legislation and institutional requirements. The patients/participants provided their written informed consent to participate in this study. Written informed consent was obtained from the individual(s) for the publication of any potentially identifiable images or data included in this article.

## Author Contributions

HL, DZ, and XZ contributed to the data collection. XZ and XC contributed to the paper assessment. DZ and GW contributed to the manuscript writing. CX, BY, and LP contributed to the formal analysis. JC contributed to the manuscript revising. All authors contributed to the article and approved the submitted version.

## Conflict of Interest

DZ and GW were employed by the company 3D Medicines Inc. Shanghai, P.R. China. The remaining authors declare that the research was conducted in the absence of any commercial or financial relationships that could be construed as a potential conflict of interest.
